# Mild and Moderate Hemophilia A: Neglected Conditions, Still with Unmet Needs

**DOI:** 10.3390/jcm12041368

**Published:** 2023-02-08

**Authors:** Giancarlo Castaman, Flora Peyvandi, Raimondo De Cristofaro, Berardino Pollio, Dario M. N. Di Minno

**Affiliations:** 1 Centre for Bleeding Disorders and Coagulation, Careggi University Hospital, 50134 Florence, Italy; 2 Complex Operative Unit of General Medicine—Hemostasis and Thrombosis, IRCCS Ca’ Granda Foundation Ospedale Maggiore Policlinico, 20122 Milan, Italy; 3 Bleeding and Thrombosis Center, Department of Medical Sciences, Agostino Gemelli University Polyclinic Foundation, 00168 Rome, Italy; 4 SIMT Immunohematology and Transfusion Medicine Service, Diagnostic Department, A.O. Città della Salute e della Scienza, 10126 Turin, Italy; 5 Department of Clinical Medicine and Surgery, Federico II University of Naples, 80131 Naples, Italy

## 1. Introduction

Hemophilia A (HA) is an inherited X-linked bleeding disorder, caused by the deficiency of coagulation factor VIII (FVIII), with variable clinical phenotypes. In addition to the risk of bleeding in all body districts, the disease is characterized by the tendency to recurrent joint bleedings, which eventually cause hemophilic arthropathy in the absence of appropriate treatment. Traditionally, the severity levels of HA are defined according to the residual plasma levels of FVIII activity. This classification system, which is commonly used and has been adopted as the standard since 2001, suggests that a residual plasma FVIII level above 40% of the predicted value can be considered as the cut-off point of normality and that, below this value, coagulation activity is insufficient [1]. Based on the amount of coagulation FVIII present, it is therefore possible to distinguish haemophilia into:

Mild: the amount of FVIII is between 6% and 40% of normal. Spontaneous bleeding is rare in this case and is often the result of trauma or surgery. Usually, patients with mild haemophilia have no functional limitations and are treated on demand.

Moderate: the amount of FVIII is very low, between 1% and 5% of the normal amount. The consequences of this condition are evident and the patient experiences extensive haematomas and prolonged bleeding and joint bleeding (hemarthrosis), even with minor trauma, resulting in pain and joint stiffness.

Severe: the level of FVIII is less than 1% of normal. This form is characterized by a high bleeding tendency with frequent joint and muscle bleeds that are mainly spontaneous. Repeated joint bleeding and subsequent arthropathy degenerate into irreversible functional impairment and result in a frequent need for orthopedic surgery [2].

Although this classification, based solely on residual FVIII levels and not on the clinical manifestation of disease, often presents a direct proportionality and correlation between these two parameters, there is nevertheless a certain phenotypic heterogeneity that render this approach systematically inapplicable. Indeed, it is not uncommon to see a subset of mild and especially moderate patients who, despite partial FVIII deficiency, present a bleeding phenotype overlapping with the symptoms of a severe disease and are therefore potentially eligible for prophylactic treatment. Unlike in cases of patients with severe hemophilia, for whom prophylaxis is the current standard of care used to prevent bleeding and attenuate the risk of arthropathy, this approach is infrequently deployed in mild and moderate patients and a significant proportion of cases may present clinical evidence of joint disease at an adult age. Clinical awareness of these patients is generally weak and prophylaxis, when adopted, may be started later compared to cases of patients with severe factor deficiency. Undoubtedly, not all mild or moderate cases present a clinical severity typical of the severe form. As such, guidance on the most appropriate treatment for these patients is still not fully established.

With this as a background, a group of Italian physicians working in the treatment of hemophilia agreed to discuss the unmet needs of patients with mild and moderate HA.

## 2. Clinical Approach by Target Population: Unresolved Issues in Mild and Moderate Hemophilia A Patients

The predictive value of residual FVIII levels in defining the severity of hemophilia has been questioned by a consensus produced by an ISTH (International Society on Thrombosis and Haemostasis) working group that brought together numerous hemophilia experts [3]. The various experiences and observations from a real-world setting suggest that this classification may, in some cases, be inadequate in the management of hemophilia patients. Therefore, treatment strategies should instead be based more on a prognostically and clinically relevant unmet need (‘clinically severe hemophilia’). In detail, the working group’s conclusions show that, in 70% of cases, the hemorrhagic phenotype has a direct correlation with the residual level of factor VIII activity; however, in the remaining 30%, the phenotype is potentially related to other individual idiopathic variables. Additionally, it is precisely in this cohort with a moderately severe form of the disease that an early implementation of prophylaxis could reduce the observation period of the natural history of the disease. It should be always borne in mind that patients with significantly different deficiency are included (FVIII 1 to 5%) within the moderate group. As such, the clinical phenotype may vary significantly.

At present, there is very limited information available in the literature that clearly and systematically describes the clinical condition, care needs, and therapeutic outcomes of patients with mild-to-moderate HA. Among the most significant studies aimed at defining the bleeding pattern, joint health, quality of life and the impact of HA on the daily life of mild-to-moderate patients, an analysis conducted in the Netherlands several years ago can be mentioned, in which an exploratory survey of 1066 haemophilic patients (16% moderate, 44% severe and 39% mild) was performed [4]. The majority of patients with moderate hemophilia (73%) reported bleeding in the previous year and a considerable proportion of these reported joint impairment (43%), chronic pain (15%), need for orthopedic aids (24%) and were unemployed due to disability (27%). A quarter of the patients with moderate HA showed a more severe hemorrhagic phenotype despite intermittent use of prophylaxis, reporting frequent bleeding, a median of eight bleedings per year (including two joint bleedings) and joint impairment (68% of cases). In conclusion, moderate hemophilic patients reported a significant burden of disease and more than 25% were on a long-term prophylaxis regimen, implemented specifically because of their frequent bleeding.

Further confirmation on the tendency of some patients with moderate hemophilia to present clinical outcomes overlapping with patients with the severe form of the disorder was provided by a recent systematic review conducted by Di Minno et al. [5]^.^ This included 31 published studies, in which the prevalence of hemophilic arthropathy ranged from 15 to 77% in patients with moderate hemophilia, and prophylactic replacement therapy was prescribed in approximately 30% of these patients, generally following a diagnosis of established arthropathy. These data confirm that the severity of hemophilia should not be defined solely by FVIII levels and that a significant proportion of patients with non-severe hemophilia could benefit from ‘early prophylaxis’. The need for initiating early prophylaxis in this population is further supported by a recent report by the DYNAMO study [6]. In this study, the median age at first joint bleed was 7 years (IQR, 4–15 years) in 70 moderate hemophilia patients and 13 years (IQR 7–20 years) in 234 mild hemophilia patients, a figure comparable to that used three previous studies (median 6.7 and 5 years in moderate and 14.2 years in mild hemophilia) [7,8,9]. 

Based on what they saw in their clinical practice, the clinicians expressed a consensus opinion regarding the unmet needs reported in the above-mentioned papers regarding the mild and moderate population and the percentage of patients who are candidates for prophylaxis. In their experience, this comprises about 30% of moderate patients and a very small percentage of mild patients. 

In this regard, clinicians point out that the international guidelines [10,11] in place for the management of haemophilia themselves suggest that the prophylaxis regimen is the standard of care, not only for people with severe hemophilia, but also for a proportion of individuals with moderate haemophilia or another congenital bleeding disorder associated with a severe hemorrhagic phenotype and/or a high risk of spontaneous bleeding.

This approach is also in line with the new definition of prophylaxis proposed by the World Federation of Hemophilia and the well-established knowledge that a trough level of protection below 1 IU/dL (1%) should now be considered an inadequate target. To date, most physicians prefer to aim for higher levels of protection (trough level >3–5% or higher, up to 10–15%). Therefore, even moderate patients, and in some circumstances mild patients with residual physiological FVIII levels below these minimum therapeutic cut-offs, are potentially candidates for prophylaxis treatment to ensure complete and safe haemostasis and minimize the related impact of the disease. Furthermore, the intrinsic limitations of current laboratory methods with their wide variance can lead to misclassification at diagnosis for subjects with factor VIII values of between 1 and 3%. Aiming at levels >1% may thus be difficult from the point of view of laboratory evaluation.

In general and in line with the ISTH consensus, the value of residual FVIII levels, although it still represents a determining and prognostic parameter for the bleeding phenotype in 70% of subjects and is the only one objectively measurable in clinical practice, includes a window that is too wide (between 1% and 5%) to be adequately predictive of the haemorrhagic phenotype for patients with moderate severity. The framing also becomes even more complex in view of the possible co-presence of various prognostic factors (e.g., blood group, thrombocytopenia, von Willebrand factor levels, *F8* or epistatic polymorphisms, etc.) that may influence the course of moderate patients.

In conclusion, clinical characteristics often do not correlate with the laboratory parameters that define the cut-offs for the categories of disease severity (mild, moderate and severe patients). Therefore, it would make sense for researchers to implement a disease severity classification that is not limited to the value of plasma factor VIII levels alone, but which also takes into account the patient’s clinical assessment. This would allow clinicians to mitigate the risk of not administering inadequate treatment or delaying the start of treatment in patients with mild or moderate disease and in patients who are also prone to significant clinical events and related complications.

In the face of wide inter-individual variability, the therapeutic approach for patients belonging to the same severity category does not necessarily need to be uniform and standardized. Rather, it may assessed on an ad hoc basis, taking into account not only the severity of haemophilia but also the clinical course (with a focus on age, type, frequency and bleeding sites). 

## 3. Important Parameters for Identifying Mild and Moderate Patients Eligible for Prophylaxis

Based on the clinical experience of the board’s experts and based on what is described in the literature, the most relevant clinical parameters and symptoms to be evaluated to determine the need for prophylaxis in a patient with mild or moderate HA include:

- Assessment of onset of first spontaneous bleeding, approximately before 6 months of age;
- Assessment of onset of first spontaneous joint bleeding, approximately before 2 years of age;
- Spontaneous bleeding at critical sites (e.g., muscular, intracranial, subcutaneous major bleeding);
- Haemorrhagic phenotype and number of bleedings per year: for patients not treated with prophylaxis, 10 bleedings/year or 5 bleedings/year, including traumatic bleedings, should prompt the clinician to consider starting prophylaxis; in the case of traumatic bleedings, the limit may be further reduced to 1 if bleeding occurs at the joint or a critical muscle site; in general, patients with ≥2 spontaneous bleedings/year should be proposed for prophylaxis;
- Underlying cause of bleeding (whether traumatic or spontaneous in nature);
- Early signs of joint degeneration (presence of synovitis) on ultrasound monitoring;
- Presence of risk factors (e.g., obesity, reduced physical activity, lower occupational and social inclusion);
- Presence of co-morbidities (e.g., oncological, cardiovascular diseases, HIV/HCV positivity);
- Patient expectations (based on the desired lifestyle and daily activities).

Regarding the identification of these parameters, therefore, the working group shares further considerations and reflections:

- Prophylaxis starts only after an established degenerative joint impairment is considered late and therefore an inappropriate criterion. Instead, it would be desirable to implement more accurate joint condition monitoring than is commonly done for mild-to-moderate patients, who undergo these instrumental examinations rarely and less frequently than necessary.
- Beginning prophylaxis at an early age would allow for the appropriate management of children and adolescents (who generally have healthy joints), in whom the chances of protection with adequate prophylaxis are much greater than in adults. With non-replacement prophylaxis, the implementation of a prophylaxis regimen at an earlier age is objectively more feasible and impactful. The recent data from a HAVEN 6 study in mild and moderate hemophilia A patients showed that only about half of the patients were on prophylaxis before starting emicizumab, a factor mainly due to the low propensity for replacement treatment among some of the moderate patients (a trend that could be changed with the availability of a treatment administered subcutaneously and less frequently) [12]. Of note, at enrolment 41/72 (57%) of patients had history of frequent bleeds and 32 out of 72 (44%) of frequent joint bleedings. In this study, prophylaxis with emicizumab was safe and efficacious. As such, all-bleed annualized bleed rates dropped from 10.1 (95% CI 6.93–14.76) at 24 weeks pre-study to 2.3 (95% CI 1.67–3.12) on study after a median follow-up of 55.6 weeks [12]
- The criteria defined above for moderate hemophilic patients can certainly also be applied to mild ones, although the incidence is substantially lower. Even in mild cases, the pattern of onset of bleeding together with clinical and radiological data of musculoskeletal impairment should prompt prophylaxis. Therefore, a clinical and holistic assessment of the patient is recommended, regardless of the level of severity defined by the laboratory parameters.

## 4. Conclusions

There is no doubt that a significant proportion of patients with moderate HA, as well as a small minority with the mild form, would benefit from the use of prophylaxis. This approach, however, should resemble the form adopted for the severe cases and implementation should probably occur earlier than is usually considered appropriate. The advent of non-replacement treatment may render this easier approach, especially in pediatric patients, and be more attractive also for reluctant adult patients who bleed only on several occasions during a year but who endure a significant long-term burden from joint health. Further controlled studies of these populations, employing novel e are required to better appreciate better the clinical impact and resulting quality of life, with novel strategies in this regard.
